# The relationship between lower limb alignment and physical fitness in children aged 10–12: A sex-specific analysis using the ALPHA-fitness test battery

**DOI:** 10.1016/j.bjpt.2025.101242

**Published:** 2025-07-14

**Authors:** Carlos Lahoz, Jorge Pérez-Rey, David González, José Gallart, Javier Bayod, María José Luesma

**Affiliations:** a Department of Education of the Government of Navarra, Santos Justo y Pastor Public School, Fustiñana, Navarra, Spain; b Department of Human Anatomy and Histology, University of Zaragoza, Calle Domingo Miral s/n, 50009, Zaragoza, Spain; c Private Practice, Gallart & González Podiatric Clinic, Calle de la Moreria, Pl. de Miguel Salamero, 4, 50004, Zaragoza, Spain; d Department of Mechanical Engineering, University of Zaragoza, 50009, Zaragoza, Spain

**Keywords:** Body mass index, Body posture, Growth and development, Paediatrics, Physical performance

## Abstract

**Background:**

Physical fitness predicts health outcomes during childhood and later life. While the influence of obesity on physical fitness is well established, the impact of lower limb alignment (such as knee alignment, foot posture, and plantar footprint) on physical fitness has been less explored.

**Objective:**

To evaluate the relationship between lower limb alignment, body mass index (BMI), and physical fitness in children aged 10–12 with a sex-specific analysis.

**Methods:**

Fifty-nine children (33 girls, 26 boys) participated in this cross-sectional study. Knee alignment (genu valgum, normal), foot posture (using FPI-6), and plantar footprint (classified by the Hernández-Corvo method) were measured. BMI was calculated to classify children into underweight, normal weight, overweight, and obese. Physical fitness was assessed using the ALPHA-Fitness Test Battery, measuring cardiorespiratory fitness, muscular strength, and speed/agility. Multiple linear regression models were developed to analyse the associations between variables.

**Results:**

Sex and BMI were significant predictors of muscular and cardiorespiratory fitness, with boys presenting better muscular and cardiorespiratory fitness. Larger BMI was associated with poorer muscular fitness (*p* = 0.003). Knee alignment in the frontal plane was not a predictive factor in physical fitness. Pronated foot was a weak predictor of lesser muscular fitness (*p* = 0.041). Plantar footprint did not significantly predict physical fitness.

**Conclusions:**

Sex and BMI were the main determinants of physical fitness. Despite some musculoskeletal factors having some influence, their relationship with physical fitness is not as direct as sex and BMI. Clinical practice should prioritise weight control in the interventions for physical fitness in children, considering sex differences.

## Highligths

• Knee misalignment affects physical fitness in children aged 10–12 years.
• Foot position affects physical fitness in children aged 10–12 years.
• Plantar footprint type affects physical fitness in children aged 10–12 years.

## Introduction

Physical fitness is a person's ability to perform physical activity, which helps study characteristics associated with a lower risk of chronic disease and premature death.^1^ Scientific evidence shows that high levels of aerobic capacity in childhood and adolescence are associated with a better cardiovascular profile in adulthood.^2^^,^^3^ Improvements in muscular strength from childhood through adolescence are associated with lower adiposity. A healthier body composition is linked to improved cardiovascular profiles in adulthood and reduced risk of premature mortality. Furthermore, optimal aerobic capacity is associated with decreased risk of overweight/obesity and metabolic risk.^4^ Sex differences significantly affect physical fitness, particularly in developing children.^5^

Poor posture can lead to pain and limited mobility,^6^ causing children to prefer sedentary activities (e.g., watching television or playing video games).^7^ These children may avoid regular participation in physical activities, resulting in a sedentary lifestyle that further exacerbates obesity, hinders engagement in exercise, and negatively impacts health status in adulthood.^8^

Obesity has a high global prevalence and poses a significant health risk for children. Research in pediatric populations has established a link between obesity and conditions such as genu valgum (knock knees) and flat feet (pes planus).^9^^,^^10^ These musculoskeletal alterations may lead to long-term complications in adulthood, particularly osteoarthritis.^9^

Despite the importance of the relationship between physical fitness in childhood and health in adulthood, few studies have linked body posture, physical fitness, and body status measured by the body mass index (BMI), and those that do exist relate only to the position of the knee (especially genu valgum) and not to the knee, foot, and plantar footprint as a whole.^11^, ^12^, ^13^, ^14^ To the best of our knowledge at the time of this study, our research is the first to incorporate the foot into postural evaluation and link it to obesity and physical fitness, allowing us to explore their interrelationship for the first time.

Understanding the circle of interconnected factors is crucial to developing effective interventions to improve children's physical health and prevent long-term consequences.^15^ This study aimed to analyse the relationship between body posture (knee position in the frontal plane, foot position, and type of plantar footprint) and BMI with physical fitness in a population of children aged between 10 and 12 years old and to analyse these associations by sex.

## Methods

An observational, descriptive, cross-sectional study was designed to achieve the proposed objective. Children were recruited from the fifth and sixth grades of the Primary School of CPEIP San Bartolomé (Navarre, Spain). The inclusion criteria were children between 10 and 12 years old. Exclusion criteria were children with physical impairments that prevented them from taking the physical fitness tests and lower limb length differences greater than 2 cm, considering that the issue can significantly impact physical performance. Nonetheless, all children could be included in the study. A total of 59 children were included.

The principles of the Declaration of Helsinki were followed. The protocol was verified and approved by the Research Ethics Committee of the Community of Aragon (CEICA) (Registration n°: PI20/263), and the legal guardians of the children signed the informed consent.

Sex, age, weight, height, body posture and physical fitness were assessed. To ensure that any observed relationship between lower limb alignment and physical fitness was accurately characterised for both boys and girls, all analyses were stratified by sex.

The physical education teacher recorded the participant's age, weight (kg), and height (m) using standardised methods and conducted physical fitness tests. For this purpose, the ALPHA-Fitness Test Battery was used in its high-priority version 21. This has previously demonstrated high reliability and validity for children and adolescents assessing muscular fitness, cardiorespiratory fitness, motor fitness, and BMI.^1^

Muscular fitness was assessed by the horizontal jump test length (cm), using the feet together.^16^ Cardiorespiratory fitness was assessed by means of the number of completed periods in the 20 m Shuttle Run Test (Course/Navette).^17^ For motor fitness, the 4 × 10 m speed/agility test proposed in the extended version was used because the present study aimed to analyse the alterations of the lower limbs concerning physical fitness. The time in seconds (s) taken to complete that distance was recorded.^18^

BMI is defined as the weight in kilograms divided by the square of height in meters (kg/m²), determining the participants' weight status (normal weight, overweight, and obese).^7^

Variables concerning body posture, such as knee alignment, foot position, and type of plantar footprint, were recorded. These measurements were taken by an independent podiatrist trained in biomechanics and paediatric podiatry, unaware of the participants' fitness test results. They were performed with the individual standing on a methacrylate podoscope at the school sports facilities during school hours.

The knee is considered to reach alignment with the mechanical axis of the lower limb at eight years of age, where the intercondylar distance (ICD) is 0 cm (±3 cm).^9^ Knee alignment was assessed with the knees in extension and neutral rotation, and the two legs were brought together so that either the femoral condyles or the tibial malleoli touched. It was measured with a tape measure expressed in cm. If, when the participant brought their legs together, the knees touched before the malleoli, the inter-malleolar distance (IMD) was measured. If, on the other hand, the malleoli touched first, the ICD was measured. This methodology has been validated in previous studies with a reported standard error between 0.5 cm and 1 cm.^9^^,^^19^

Participants with IMD greater than or equal to 4 cm were classified as genu valgum, genu varum for ICD values greater than or equal to 3 cm, and genu normal for IMD values <4 cm and ICD <3 cm.^19^^,^^19^

To assess the foot position, the children were asked to walk a few steps and then stand still,^20^ then their feet were classified as supinated, neutral, or pronated, using the Foot Posture Index (FPI-6).^21^

The plantar footprint was assessed with an ink pedograph in which each subject, in an orthostatic position, left their footprints printed on a piece of paper.^22^ Determination of the footprint type was carried out according to the Hernández Corvo method,^23^^,^^24^ classifying the plantar footprint into flat, normal, and cavus.^22^^,^^24^

Data were collected in an Excel spreadsheet (Microsoft Office Professional Plus 2016, Microsoft Inc., USA). IBM SPSS software version 24 (SPSS®IBM® Corporation, New York, USA) was used for data analysis. The mean of the results obtained in each participant's physical fitness test (dependent variables) was compared to the different characteristics of the lower limbs analysed and broken down by sex. To study the relationship between physical fitness, knee position, FPI, and plantar footprint by sex, mean (SD) and 95 % CI were calculated.

To quantify the relation between independent variables (sex, BMI, knee position, FPI, and plantar footprint) with dependent variables (muscular fitness, motor fitness, and cardiorespiratory fitness) multiple linear regression models were developed.

## Results

When BMI was analysed by sex, no underweight boys were found according to the WHO tables.^7^46.2 % had a normal weight, 23.1 % were overweight, and 30.8 % were obese. 6.1 % of girls were underweight, 54.5 % had a normal weight, 24.2 % were overweight, and 15.2 % were obese.

Of the 59 students, 33 were girls, and 26 were boys, with a mean age of 11.50 years for the boys’ group and 11.48 for the girls. Significant variability was found in variables such as weight, height, and BMI. ICD in the group of girls was 0, demonstrating that they all had a genu valgum or normal. In the group of boys, a small IMD distance was measured in some cases (Table 1).

**Table 1:** Sample characterisation.

Variables		
Students (*n* = 59)	Boys (*n* = 26) Mean (SD) [95 % CI]	Girls (*n* = 33) Mean (SD) [95 % CI]
Age (years)	11.50 (0.66) [11.32, 11.69]	11.48 (0.66) [11.32, 11.65]
Weight (kg)	46.26 (12.51) [44.02, 50.92]	43.50 (13.77) [41.04, 47.76]
Height (m)	1.49 (0.08) [1.47, 1.52]	1.47 (0.09) [1.45, 1.49]
BMI (kg/m^2^)	20.84 (3.78) [19.81, 21.89]	20.13 (4.65) [18.99, 21.27]
IMD (cm)	2.76 (2.75) [2.01, 3.53]	2.61 (2.29) [2.06, 3.18]
ICD (cm)	0.32 (0.92) [0.07, 0.58]	0.0 (0.0) [——–]
FPI	2.56 (4.41) [1.33, 3.79]	3.35 (4.19) [2.32, 4.38]
Muscular fitness (cm)	150.0 (18.3) [144.9, 155.1]	134.0 (18.6) [129.4, 138.6]
Motor fitness (s)	12.4 (1.6) [11.9, 12.8]	13.0 (1.7) [12.6, 13.4]
Cardiorespiratory fitness (periods)	8.3 (3.2) [7.4, 9.2]	6.7 (2.5) [6.1, 7.3]

Data for physical fitness test and knee alignment are provided in Table 2. Table 3 presents the outcomes of the physical fitness tests based on foot alignment as assessed with the FPI-6. Table 4 shows the results of the physical fitness tests and their association with the type of plantar footprint.

**Table 2:** Physical fitness test results about knee alignment and their statistical significance.

	Knee position	Boys Mean (SD) [95 %CI]	Girls Mean (SD) [95 %CI]
Muscular fitness (cm)	Genu valgum	139.8 (17.4) [127.3, 152.3]	127.0 (23.3) [113.6, 140.4]
	Genu normal	152.4 (17.9) [146.9, 158.0]	135.9 (16.8) [131.2, 140.6]
Motor fitness (s)	Genu valgum	13.5 (1.5) [12.4, 14.5]	13.1 (1.1) [12.4, 13.7]
	Genu normal	12.1 (1.5) [11.7, 12.6]	13.0 (1.8) [12.5, 13.5]
Cardioresp. fitness (periods)	Genu valgum	5.6 (1.3) [4.7, 6.5]	5.8 (2.3) [4.5, 7.1]
	Genu normal	8.9 (3.2) [7.9, 9.9]	6.9 (2.5) [6.3, 7.6]

**Table 3:** Physical fitness test results in relation to FPI-6.

	FPI-6	Boys Mean (SD) [95 %CI]	Girls Mean (SD) [95 %CI]
Muscular fitness (cm)	Supinated foot	147.3 (15.6) [135.4, 159.3]	130.0 (17.0) [118.6, 141.4]
	Neutral foot	152.7 (21.1) [144.5, 160.9]	139.7 (18.0) [133.2, 146.3]
	Pronated foot	146.5 (13.8) [138.9, 154.2]	128.4 (18.5) [120.6, 136.2]
Motor fitness (s)	Supinated foot	10.9 (1.1) [10.0, 11.7]	13.3 (2.4) [11.7, 14.9]
	Neutral foot	12.6 (1.5) [12.0, 13.1]	12.9 (1.3) [12.4, 13.3]
	Pronated foot	13.0 (1.6) [12.2, 13.9]	13.0 (1.8) [12.3, 13.8]
Cardioresp. fitness (periods)	Supinated foot	10.1 (2.9) [7.9, 12.4]	7.1 (2.1) [5.6, 8.5]
	Neutral foot	8.5 (3.0) [7.3, 9.6]	7.0 (2.2) [6.2, 7.8]
	Pronated foot	6.8 (3.4) [5.0, 8.7]	6.2 (2.9) [4.9, 7.4]

**Table 4:** Physical fitness test results in relation to plantar footprint analysis.

	Plantar footprint	Boys Mean (SD) [95 %CI]	Girls Mean (SD) [95 %CI]
Muscular fitness (cm)	Flat	147.4 (18.7) [124.2, 170.6]	122.0 (32.4) [88.0, 156.0]
	Normal	146.5 (19.5) [138.3, 154.7]	139.3 (13.4) [132.7, 146.0]
	Cavus	154.2 (16.8) [146.9, 161.6]	133.4 (17.6) [128.0, 138.9]
Motor fitness (s)	Flat	12.1 (0.8) [11.1, 13.2]	14.4 (1.9) [12.5, 16.4]
	Normal	12.7 (1.6) [12.0, 13.4]	12.6 (1.1) [12.1, 13.1]
	Cavus	12.1 (1.7) [11.4, 12.9]	13.0 (1.8) [12.4, 13.5]
Cardioresp. fitness (periods)	Flat	7.4 (3.6) [2.9, 11.9]	3.3 (1.3) [2.0, 4.7]
	Normal	7.3 (3.5) [5.8, 8.8]	7.3 (2.3) [6.2, 8.5]
	Cavus	9.5 (2.5) [8.4, 10.6]	6.9 (2.4) [6.2, 7.6]

In the multiple linear regression models (Table 5), for muscular fitness, it was found that sex (*p* < 0.001), BMI (*p* = 0.003), and FPI (pronated foot, *p* = 0.041) had a significant influence. In motor fitness, only sex was significant (*p* = 0.049), and in cardiorespiratory fitness, sex (*p* < 0.001) and BMI (*p* = 0.015).

**Table 5:** Multiple linear regression models for physical fitness components.

Muscular fitness (cm)	95 % CI for B							
Independent variables	B	SE	Beta	t	Sig.	Lower	Upper	R^2^
Constant	182,00	9.31		19.55	<0.001	163.55	200.45	0.262
Sex^1^	−16.20	3.29	−0.40	−4.92	<0.001	−22.73	−9.67	
BMI	−1.30	0.43	−0.28	−3.04	0.003	−2.15	−0.45	
Knee position^2^	−4.16	4.45	−0.08	−0.93	0.352	−12.98	4.66	
FPI supinated^3^	−8.74	4.49	−0.16	−1.95	0.054	−17.65	0.16	
FPI pronated^3^	−8.00	3.86	−0.19	−2.07	0.041	−15.66	−0.35	
Plantar footprint Flat^4^	0.48	6.33	0.01	0.08	0.939	−12.07	13.03	
Plantar footprint Cavus^4^	−0.56	3.55	−0.01	−0.16	0.875	−7.59	6.48	
Motor fitness								
Constant	11.16	0.88		12.72	<0.001	9.42	12.90	0.038
Sex^1^	0.62	0.31	0.19	1.99	0.049	0.00	1.23	
BMI	0.06	0.04	0.15	1.44	0.153	−0.02	0.14	
Knee position^2^	0.21	0.42	0.05	0.51	0.614	−0.62	1.04	
FPI supinated^3^	−0.48	0.42	−0.11	−1.12	0.263	−1.32	0.36	
FPI pronated^3^	0.14	0.36	0.04	0.39	0.696	−0.58	0.86	
Plantar footprint Flat^4^	0.27	0.60	0.05	0.46	0.647	−0.91	1.46	
Plantar footprint Cavus^4^	0.01	0.33	0.01	0.01	0.993	−0.66	0.67	
Cardiorespiratory fitness								
Constant	11.73	1.41		8.33	<0.001	8.94	14.51	0.213
Sex^1^	−1.73	0.50	−0.29	−3.48	<0.001	−2.72	−0.75	
BMI	−0.16	0.06	−0.23	−2.48	0.015	−0.29	−0.03	
Knee position^2^	−0.94	0.67	−0.13	−1.40	0.165	−2.27	0.39	
FPI supinated^3^	0.50	0.68	0.06	0.74	0.461	−0.84	1.85	
FPI pronated^3^	−0.61	0.58	−0.10	−1.05	0.295	−1.77	0.55	
Plantar footprint Flat^4^	−0.79	0.96	−0.08	−0.83	0.410	−2.69	1.11	
Plantar footprint Cavus^4^	0.56	0.54	0.10	1.04	0.299	−0.50	1.62	

Reference category: ^1^Boys; ^2^Normal; ^3^Neutral; ^4^Normal.

## Discussion

Boys showed differences in muscular, motor, and cardiorespiratory fitness compared to girls. Descriptive data suggested poorer performance in boys with genu valgum, pronated foot, or obesity. However, regression analysis revealed sex and BMI were stronger predictors of fitness components. For girls, flat plantar footprint and obesity correlated with worse cardiorespiratory fitness in descriptive analysis; however, plantar footprint type's significance as a predictor varied in regression models. Underweight or obese girls displayed lower muscular and motor fitness.

Aligned with previous findings,^4^^,^^5^ our study's regression analysis confirmed sex as a significant predictor of muscular fitness (*p* < 0.001), cardiorespiratory fitness (*p* < 0.001), and motor fitness (*p* = 0.049). While some research notes genu varum prevalence,^25^ we lacked participants with this feature, only having participants with genu valgum/neutral knee positions.

Overall BMI distribution was similar between sexes, with higher obesity in boys (30.8 %) than girls (15.2 %), consistent with global trends^26^ and exceeding typical reports.^27^ Cardiorespiratory fitness inversely related to BMI, as seen in the study by Ruiz et al.^28^ Regression analysis showed BMI significantly predicted muscular fitness (*p* = 0.003) and showed a trend towards significance for cardiorespiratory fitness (*p* = 0.015). Literature suggests higher flat feet prevalence in obese children, without direct fitness implication.^29^

In boys, descriptive data suggested genu valgum negatively affected motor and cardiorespiratory fitness. However, regression analysis, using significance values, did not find knee position to be a significant independent predictor of any fitness component (*p* > 0.165). While Molina et al.^11^ link physical performance to posture over obesity, and other authors^10^^,^^30^ associate genu valgum with impaired balance/stability, our multivariate analysis did not confirm knee alignment as a significant independent factor.

Descriptive data showed better motor and cardiorespiratory fitness with supinated feet compared to pronated. Yet, regression analysis indicated FPI's limited significance. Specifically, FPI pronated was a significant predictor of muscular strength (*p* = 0.041), but FPI supinated was not (*p* = 0.054). FPI was not a significant predictor of motor or cardiorespiratory fitness. This contrasts with reports^31^^,^^32^ of pronated feet linking to fatigue/pain and increased joint loading, suggesting this association holds for muscular strength but the relationship is more complex regarding other fitness measures. Plantar footprint type did not significantly affect boys' physical fitness in regression models. Given influences of foot position/BMI on footprint type, current research emphasizes foot position in fitness studies.^33^ Dowling et al.^16^ noted that flat footprints' increased joint loading doesn't always translate to poorer dynamic fitness, consistent with our findings.

Literature strongly links excess weight to impaired aerobic capacity and motor coordination^16^ and altered running patterns.^34^ Our study supports BMI's significant prediction of muscular fitness (*p* = 0.003) and trend for cardiorespiratory fitness (*p* = 0.015). Obese boys showed non-significant differences in jumping distance (muscular fitness) compared to peers, aligning with challenges defining the obesity-muscular fitness link.^35^ While obesity might increase muscular strength due to higher muscle mass, weight-bearing activities like jumping could be negatively impacted. Regression analysis confirmed a significant adverse effect of BMI on muscular strength (*p* = 0.003). Descriptive data indicated that obese boys had lower motor skills than normal/overweight peers, but no difference between normal/overweight. Obesity impairs motor fitness,^36^ but regression analysis did not confirm BMI as a significant predictor. In girls, knee/foot position did not significantly affect physical fitness, consistent with Molina-Garcia et al.,^11^ suggesting body weight/activity levels are more influential than posture. This aligns with our regression analysis findings.

Shin et al.^37^ showed flat feet affect foot/ankle kinematics, and foot position influences gait,^31^ with flat feet linked to altered ankle/knee/hip kinematics.^38^ While our descriptive data showed fitness differences related to foot posture/footprint in girls, regression models did not confirm significance. Pronated feet might reduce physical activity and fitness,^15^ but our results suggest a more intricate relationship. Girls with flat footprints had worse cardiorespiratory fitness, consistent with Shin et al.^37^ link between flatfoot deformity and limited aerobic efficiency. However, plantar footprint was not a significant predictor in our regression model.

Underweight/obese girls had poorer muscular fitness, aligning with studies on the adverse effects of extreme BMIs,^16^ possibly more pronounced in girls due to muscle mass distribution.^39^ Normal/overweight girls had better motor fitness, consistent with links between extreme body composition and impaired motor function,^40^ and regression analysis supports obesity's negative impact on muscular fitness (*p* = 0.003). While some studies suggest reduced fitness in underweight individuals,^41^ we found no significant differences, with obesity as the main factor impairing cardiorespiratory fitness.^42^ Our study underscores BMI's role, though regression analysis indicates a weaker influence on motor fitness than descriptive data implied.

The limitations of our study included the age of the participants, who were undergoing rapid physical development with constant changes in skeletal and muscle mass, which can influence values for lower limb angulation, specifically at the knee joint.^14^ Shohat et al.^19^ highlight the need to consider these developmental changes when assessing knee alignment, which can impact posture and fitness. In addition, the stages of sexual and skeletal maturation can vary significantly among children.

Our study focused on the lower limb, excluding other physical skills assessed in the ALPHA test. Future studies should apply the entire test battery, analysing a larger sample size for better evidence.

## Conclusions

In our research, sex and BMI were shown to be determinants of physical fitness, with boys obtaining better results in muscular and cardiorespiratory fitness tests. BMI had a significant inverse relationship with muscular fitness and a tendency to predict cardiorespiratory fitness. In multiple linear regression analyses, knee alignment did not independently predict any component of fitness, and foot posture had a limited predictive role, showing mainly a weak association of pronated feet with decreased muscular strength. This implies that the influence of these musculoskeletal characteristics may be less direct and mediated by other factors, with sex and BMI playing more dominant roles. These findings underscore the clinical importance of addressing weight control in interventions to improve children's fitness and take into account sex-specific differences.

## Declaration of competing interest

The authors declare no competing interest.
